# Amygdala activity after subchronic escitalopram administration in healthy volunteers: A pharmaco-functional magnetic resonance imaging study

**DOI:** 10.1177/02698811241286773

**Published:** 2024-10-04

**Authors:** Paulina B Lukow, Millie Lowther, Alexandra C Pike, Yumeya Yamamori, Alice V Chavanne, Siobhan Gormley, Jessica Aylward, Tayla McCloud, Talya Goble, Julia Rodriguez-Sanchez, Ella W Tuominen, Sarah K Buehler, Peter Kirk, Oliver J Robinson

**Affiliations:** 1 Institute of Cognitive Neuroscience, University College London, London, UK; 2 Department of Psychology & York Biomedical Research Institute, University of York, York, UK; 3 Université Paris-Saclay, Institut National de la Santé et de la Recherche Médicale, INSERM U1299 “Trajectoires Développementales Psychiatrie,” Ecole Normale Supérieure Paris-Saclay, CNRS UMR 9010, Centre Borelli, Gif-sur-Yvette, France; 4 Department of Psychology, Humboldt-Universität zu Berlin, Berlin, Germany; 5 UCL Division of Psychiatry, Maple House, London, UK; 6 Centre for Medical Image Computing, Department of Computer Science, University College London, London, UK; 7 Emotion and Development Branch, National Institute of Mental Health, Bethesda, MD, USA

**Keywords:** Functional magnetic resonance imaging, emotion, antidepressants, escitalopram, anxiety

## Abstract

**Background:**

Selective serotonin reuptake inhibitors (SSRIs) are used for the treatment of several conditions including anxiety disorders, but the basic neurobiology of serotonin function remains unclear. The amygdala and prefrontal cortex are strongly innervated by serotonergic projections and have been suggested to play an important role in anxiety expression. However, serotonergic function in behaviour and SSRI-mediated neurobiological changes remain incompletely understood.

**Aims:**

To investigate the neural correlates of subchronic antidepressant administration.

**Methods:**

We investigated whether the 2- to 3-week administration of a highly selective SSRI (escitalopram) would alter brain activation on a task robustly shown to recruit the bilateral amygdala and frontal cortices in a large healthy volunteer sample. Participants performed the task during a functional magnetic resonance imaging acquisition before (*n* = 96) and after subchronic escitalopram (*n* = 46, days of administration mean (SD) = 15.7 (2.70)) or placebo (*n* = 40 days of administration mean (SD) = 16.2 (2.90)) self-administration.

**Results:**

Compared to placebo, we found an elevation in right amygdala activation to the task after escitalopram administration without significant changes in mood. This effect was not seen in the left amygdala, the dorsomedial region of interest, the subgenual anterior cingulate cortex or the right fusiform area. There were no significant changes in connectivity between the dorsomedial cortex and amygdala or the subgenual anterior cingulate cortex after escitalopram administration.

**Conclusions:**

To date, this most highly powered study of subchronic SSRI administration indicates that, contrary to effects often seen in patients with anxiety disorders, subchronic SSRI treatment may *increase* amygdala activation in healthy controls. This finding highlights important gaps in our understanding of the functional role of serotonin.

## Introduction

Serotonergic neurotransmission mediates numerous brain functions, from mood to cognition (Duerler et al., 2022; Puglisi-Allegra and Andolina, 2015), and its pharmacological manipulation is used in the treatment of multiple conditions, from anxiety disorders to chronic pain. This is commonly achieved with antidepressant medications of the selective serotonin reuptake inhibitor (SSRI) class (National Institute for Health and Care Excellence, 2020). Despite the extensive use of SSRIs, serotonergic function in the brain and SSRI-mediated neurobiological changes remain incompletely understood.

Serotonergic projections innervate numerous brain regions, modulating multiple brain functions such as anxiety (Parent and Descarries, 2020; Peters et al., 2021). Anxiety and associated cognitions are crucial for threat detection in the environment, driving behaviours that protect the individual from harm. SSRIs can successfully treat persistent, pathological anxiety (Bandelow et al., 2015), indicating a key role for serotonin in anxiety processing. However, the key neurobiological mechanisms of SSRI-mediated anxiolysis are unknown. Brain areas implicated in both healthy and pathological anxiety include the amygdala and prefrontal cortex. Both their activation and connectivity are associated with anxiety (Brühl et al., 2014; Carlisi and Robinson, 2018; Chavanne and Robinson, 2021; Etkin and Wager, 2007; Freitas-Ferrari et al., 2010; Goossen et al., 2019; Hattingh et al., 2012; Ipser et al., 2013; Kalisch and Gerlicher, 2014; McTeague et al., 2020; Mochcovitch et al., 2014; Robinson et al., 2012). Both regions receive strong serotonergic projections (Parent and Descarries, 2020), and modulation of serotonin in these regions alters both anxiety (Bocchio et al., 2016) and aversive processing (Duerler et al., 2022; Garcia-Garcia and Soiza-Reilly, 2019). Moreover, acute depletion of the serotonin precursor tryptophan was found to increase anxiety-related amygdala-prefrontal connectivity (Robinson et al., 2013). Altogether, the evidence suggests that serotonergic function in the amygdala and prefrontal cortex serves an important function in anxiety and aversive processing.

The amygdala and prefrontal cortex have been frequently shown to be recruited during facial emotion-processing tasks (Davis and Whalen, 2000; Gangopadhyay et al., 2021; Phillips et al., 2003; Xu et al., 2016). In our previous research in healthy volunteers, we have used such task to show robust activation within the bilateral amygdala and the right fusiform area (rFFA), and deactivation of the subgenual anterior cingulate cortex (sgACC) (Nord et al., 2017). The activation of the amygdala and prefrontal cortex has, moreover, been shown to be modulated by SSRIs. A meta-analysis of functional magnetic resonance imaging (fMRI) studies in healthy volunteers showed a decrease in brain activation in these regions to emotional stimuli after acute (i.e., single dose) antidepressant administration (Outhred et al., 2013). The prefrontal cortex can exert regulatory control over the amygdala during emotion processing (Gangopadhyay et al., 2021; Zhang et al., 2021), and this connectivity has also been shown to be elevated by SSRIs (Outhred et al., 2015; Sladky et al., 2015). Thus, both activation and connectivity between these two areas may be altered by SSRIs to elicit their anxiolytic function. The evidence supporting this hypothesis comes largely from studies on the acute effects of SSRI intake—but the timescale of SSRI administration can be an important factor in their cognitive effects. Specifically, in healthy human volunteers, a single dose of citalopram elevates anxiety-potentiated startle (Grillon et al., 2007), while a 2-week administration of the same drug has the opposite effect: reduced anxiety-potentiated startle (Grillon et al., 2009). Thus, we sought to employ a previously used emotional face-processing task (O’Nions et al., 2011) to investigate putative changes in activation and connectivity of the amygdala-prefrontal circuit after extended (2–3 weeks) SSRI administration.

We chose the SSRI escitalopram, as it has the highest serotonin reuptake selectivity of its class (Rao, 2007). Prior work delivering escitalopram (or citalopram) over a subchronic administration period (over the 10 days necessary to achieve a steady state of the pharmacology, but less than the 4 weeks shown to be clinically effective (Jakubovski et al., 2019; Rao, 2007) found reduced, increased or no changes in amygdala activation during emotional face processing in healthy volunteers (reduced: Harmer et al., 2006; Maron et al., 2016; Windischberger et al., 2010; increased: Norbury et al., 2009; unchanged: Arce et al., 2008; Henry et al., 2013; McCabe et al., 2010; Simmons et al., 2009). These inconsistencies may be related to low statistical power, as the largest sample size in these studies was 16 participants. Thus, we sought to apply our paradigm to a substantially larger sample.

In the present study, we therefore sought to investigate whether prefrontal and amygdala activation and connectivity during an established emotional face-processing task (O’Nions et al., 2011) are modulated by escitalopram administration on a relatively large sample of healthy controls. We hypothesised that compared to placebo, escitalopram treatment would (1) reduce the activation within the left amygdala, right amygdala and the rFFA, (2) increase activation within the sgACC and (3) reduce the connectivity between the dorsomedial region of interest (ROI) and the left and right amygdala.

## Materials and methods

The study was approved by the University College London Research Ethics Committee (6198/002). All analyses were performed after the pre-registration of analysis methods (https://osf.io/bdmvp) and after the completion of data collection.

### Participants

In all, 98 participants were recruited from the general population through public advertisement between 27th November 2017 and 1st June 2022, until funding expiry. The original proposal including sample size calculation can be found at https://osf.io/u8dma. All participants were between 18 and 50 years old, fluent in English, registered with a GP and able to provide written informed consent. None had consumed alcohol within 12 h prior to the study, recently used illicit drugs, had any contraindications to MRI scanning, were pregnant or breastfeeding, had impaired or uncorrected vision or hearing or were colour-blind. All participants showed good psychiatric (assessed with the Mini International Neuropsychiatric Interview (MINI; Sheehan et al., 1998)) health, had no personal history of long-term medical conditions or psychiatric illness (including substance dependence) and no family history of mood disorder, including panic disorder. All participants provided informed written consent according to the Declaration of Helsinki (World Medical Association, 2013) and were advised that they may disengage from the study at any point without giving a reason.

### Study procedures

The study comprised a double-blind, repeated measures design. Participants completed three study visits. During the first visit (conducted at the Institute of Cognitive Neuroscience, Alexandra House, 17-19 Queen Square, London, WC1N 3AZ), a researcher from the team conducted the MINI and eligibility screening with the participant. Additionally, the participants completed the following questionnaires: The Beck Depression Inventory, Generalized Anxiety Disorder 7-item scale, The Patient Health Questionnaire 9-item scale and the State-Trait Anxiety Inventory (Beck et al., 1996; Kroenke et al., 2001; Spielberger et al., 1983; Spitzer et al., 2006). During the second study visit (within 12–23 days of the first visit), participants performed a previously used emotional face-processing task (O’Nions et al., 2011) during an fMRI acquisition. The participants were randomised to take 10 mg escitalopram or an identically appearing placebo. This dose of escitalopram is recommended for the treatment of anxiety disorders (Taylor et al., 2021), based on the evidence of escitalopram’s efficacy superior to placebo in the treatment of generalised anxiety disorder, panic disorder and social anxiety disorder in the 5–20 mg range (Baldwin et al., 2016; Blanco et al., 2013; Kong et al., 2020; Stahl and Li, 2003). In the case of generalised anxiety disorder, 10 mg has been suggested as the minimal effective dose (Davidson et al., 2010). Thus, 10 mg was chosen to employ a dose of clinical efficacy, while limiting side effects which have been found to increase with higher dosages of antidepressants (Furukawa et al., 2019). The dose was equivalent to the one used in previous studies (Arce et al., 2008; Maron et al., 2016; Simmons et al., 2009; Windischberger et al., 2010). The randomisation procedure was performed by an independent researcher before participant recruitment by pre-generating a list of group allocations with a random number generator. Researchers conducting participant visits were blind to the randomisation status. After 12–23 days of placebo or escitalopram treatment, the participants repeated the same scanning procedure as in visit two.

### Antidepressant medication

Escitalopram was self-administered by study participants for 12–23 days after their first scan. The mode of administration was a single tablet of 10 mg escitalopram, manufactured and donated for research by Lundbeck (tablet core: microcrystalline cellulose, colloidal anhydrous silica, croscarmellose sodium, talc, magnesium stearate; tablet coating: hypromellose 6cP, titanium dioxide (E171), macrogol 6000). Participants were instructed to take one tablet per day, around the same time each day, with or without food. Participants randomised to the placebo arm took a placebo matched in colour and size, also manufactured by Lundbeck. No participants took other medications or recreational drugs during escitalopram administration. Compliance with treatment was ascertained by self-report. Compliance data were available for 43 out of the 46 participants included in the analysis in the escitalopram group, and 33 out of 40 participants included in the analysis in the placebo group. The average number of days of recorded medication (escitalopram or placebo) intake was mean (SD) = 15.1 (2.51) for the escitalopram group and mean (SD) = 15.3 (2.48) for the placebo group. There was no statistical difference in compliance between the study groups (unpaired *t*-test, *p* = 0.69). Data on participant beliefs about which arm of the study they were a part of were unfortunately not collected.

### Demographic and clinical information analysis

Demographic (age and sex) and clinical information (i.e., questionnaire scores) were analysed using R version 4.2.2 using a two-sample *t*-test at follow-up.

### The emotional face-processing task

**Figure 1.**
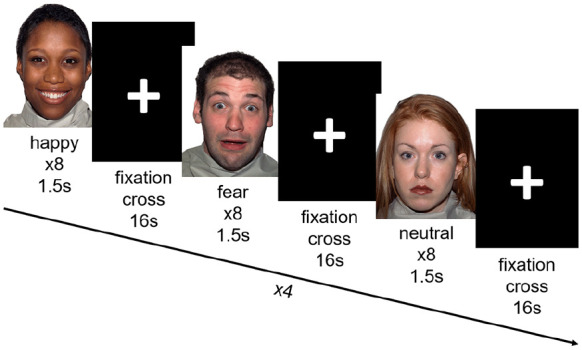
Schematic of the emotional face processing task. Participants viewed blocks of happy, fearful and neutral faces, separated by a fixation cross. There were 12 blocks of stimuli in total, and four of each emotion. Participants were instructed to label the gender of the faces displayed via button press.

The task performed by the participants during both baseline and follow-up scans was a previously used emotional face-processing task (O’Nions et al., 2011) (Figure 1). The task had a block design and 8 faces were displayed in each of the 12 blocks. The participants identified the gender of the displayed face and responded with a button press during each trial. The 12 blocks comprised happy, fearful and neutral facial expressions of 1.5 s duration. The block valence changed in an alternating fashion. The displayed stimuli were randomised for each participant and each run of the task. The displayed faces were counterbalanced for age and gender. A fixation cross was displayed for 500 ms before each face. Additionally, a rest block including the fixation cross was displayed after each face block. Both the face and rest blocks had a duration of 16 s. The total task length was 6 min 24 s. The participant’s engagement with the task was ascertained verbally through an intercom, as well as through observing the participant via a camera. No participant performed worse than chance.

### Image acquisition

All scanning was performed on a 1.5T Siemens Avanto scanner at the Birkbeck-UCL Centre for Neuroimaging, 26 Bedford Way, London, WC1H 0AP. A 32-channel head coil was used. A field map (T2*-weighted images: repetition time (TR) = 1170 ms, echo time (TE1) = 10 ms, TE2 = 14.76 ms, field of view (FOV) = 64 × 64, voxel size = 3 mm × 3 mm × 2 mm, slice thickness = 2 mm, flip angle = 90°, 64 volumes) was obtained for field map correction at pre-processing. EPI scans (T2*-weighted images: TR = 3500 ms, TE = 50 ms, FOV = 64 × 64, voxel size = 3 mm, slices = 40, slice thickness = 2 mm, flip angle = 90°, approximately 110 volumes) were collected during the task performance. An MPRAGE (T1-weighted images: TR = 2730 ms, TE = 3.57 ms, FOV = 224 × 256, voxel size = 1 mm, slice thickness = 1 mm, flip angle = 7°, 176 volumes) acquisition was run at the end of the session to generate a high-resolution structural image.

### fMRI pre-processing

Pre-processing of the fMRI data (slice time correction, motion correction, field map-based distortion correction, co-registration and normalisation) was performed with fMRIPrep version 20.2.7 (Esteban et al., 2018). All fMRIPrep options used can be found at https://fmriprep.org/en/20.2.7/workflows.html. The initial three volumes, collected during the stabilisation of the magnetic field within the scanner, were removed from the acquisition, as well as any volumes acquired after the task ended due to manual EPI acquisition stop. Subsequently, *3dBlurToFWHM, 3dTstat* and *3dcalc* from the Analysis of Functional Neuroimages (AFNI) software were used for fMRI series smoothing (Gaussian kernel: 6 mm full width at half maximum) and grand mean scaling (Chen et al., 2017) within an Montreal Neurological Institute (MNI) space-specific grey matter mask.

### Data exclusions

A visual quality assessment was performed on the fMRIPrep output. Anatomical image segmentation, normalisation of the anatomical image to the MNI152 template, field map correction of the fMRI series and the co-registration of the fMRI series onto the anatomical T1 image were visually inspected. Whole scans were excluded if more than 20% of their volumes exceeded framewise displacement of 1.3 mm.

### Within-subject model estimation for activation analyses

All within-subject activation models were estimated with AFNI 23.0.0 *3dDeconvolve* function. The contrast of interest was faces versus fixation cross across all emotion blocks. The fixation cross constituted an implicit baseline condition. Each individual-level model contained 13 movement regressors derived from fMRIPrep output (head motion in the *x, y, z*, pitch, roll and yaw directions as well as each of the parameter’s framewise derivative and a final, overall framewise displacement). Additionally, censoring of volumes exceeding framewise displacement of 1.3 mm (Siegel et al., 2014) was included in each model. De-trending of the time series was performed with the built-in option in *3dDeconvolve*.

### Within-subject model estimation for connectivity analyses

All within-subject modelling for connectivity analyses was repeated for the activation analyses. Additional regressors were included in the connectivity analyses to estimate a generalised psychophysiological interaction (gPPI) general linear model for each participant and each seed of interest (right amygdala, left amygdala and sgACC, chosen according to previous publications (Nord et al., 2017, 2019)). The activation time series was first extracted from the seeds, using the same anatomical masks employed in the activation analyses described below. The time series was then deconvolved from the canonical haemodynamic response function and resampled to match the resolution of stimulus onset times. Psychophysiological interaction terms (PPI terms) were then computed for each seed, and for each subject individually, by multiplying the deconvolved activation time series by the regressors containing onset times and durations of each stimulus type in the task (happy/fearful/neutral), that is, the psychological terms. Finally, the timeseries was re-convolved with the haemodynamic response function. The PPI terms were then entered as regressors into a general linear model containing the extracted seed time series and activation to each stimulus type, in addition to the movement and censor regressors.

### Group-level brain activation and connectivity analyses

In all analyses, the same anatomical masks were used that had been employed in our previous studies (Supplemental Figure 1 and (Nord et al., 2017, 2019)). These masks were (a) the anatomical masks defined through the PickAtlas (Nord et al., 2017, 2019) for, respectively, the right and left amygdala, (b) a custom anatomical mask for the sgACC, generated based on probabilistic maps of distinct cyto- and receptor-architectonic features of this region (Nord et al., 2017, 2019) (c) a custom mask of a dorsomedial region of interest (referred to hereafter as the dorsal ROI), based on a functional cluster resulting from a threat of shock task analysis in a previous study (Nord et al., 2019) and (d) a custom anatomical mask of the rFFA (Nord et al., 2019). The bilateral amygdala, dorsomedial frontal cortex and sgACC were the regions of interest, whereas the rFFA was the control region, given its robust activation during face-processing tasks (Fusar-Poli et al., 2009). Activation during emotional face processing was investigated for all five regions. Connectivity with the dorsal ROI was investigated for the bilateral amygdala and the sgACC.

Our primary analysis was done in R 4.2.2., adapting the same analysis approach as that taken in the publications we aimed to replicate (Nord et al., 2017, 2019). These analyses compared the average activation or connectivity parameters (i.e. the beta weights) extracted from individual subject-level models using the regional masks with AFNI’s *3dmaskave*. For baseline analyses, one-sample *t*-tests against 0 were conducted with R’s *t.test* function. For the follow-up data, equivalent analyses were conducted with R’s *lm* function, including baseline activation or connectivity maps as a covariate of no interest, a method shown to have the largest power to estimate post-treatment effects (Assmann et al., 2000; Vickers, 2001). Effect sizes were estimated in Cohen’s *d* with R’s *cohensD* function.

Secondly, we complemented each of these group-level analyses (activation and connectivity, baseline and follow-up) by running their equivalents in AFNI with small volume correction within each ROI as sensitivity analyses. Clusters of activation/connectivity resulting from these analyses were visualised in MRIcroGL 1.2.20220720. Methodological details and results of these analyses are shown in the Supplement.

Finally, we conducted two-sample *t*-tests including data resulting from the subtraction of the baseline beta-weight maps from the follow-up beta-weight maps. While present in our pre-registration protocol, this method was not used for primary inference as it was shown to be affected by measurement error and regression to the mean and to oppose the principles of appropriate randomisation, which was conducted in this study (Harrell, 2017; Knol et al., 2012; Roberts and Torgerson, 1999; Van Breukelen, 2006). We conducted these analyses both using the average activation parameters and in AFNI. Methodological details and results of these analyses are shown in the Supplement.

## Results

### Participants

From the initially recruited 98 participants, one was excluded from both baseline and follow-up analyses due to excessive movement during the baseline scan, and one more participant was excluded due to a technical issue with the recording of the parameters of the task (stimulus onset and participant responses) during fMRI acquisition at their baseline visit. The final sample size for the baseline analysis was *n* = 96. Eight participants could not be included in the follow-up analysis due to withdrawal from the study (medication side effects (*n* = 4), personal reasons (*n* = 2), misadministration of the medication (*n* = 1)) or a technical issue with the recording of task parameters (*n* = 1). A further two participants were excluded from the follow-up analysis due to excessive motion. There were no serious adverse events and the rates of side effects were low (the reported frequency of side effects was ‘absent’ at least 80% of the time on days 3, 7 and 14; for details, see Supplement). The final sample size of the intention to treat (ITT) analysis (total *n* = 86) for the follow-up analysis was *n* = 40 (placebo group, days of placebo administration mean (SD) = 16.2 (2.90)) and *n* = 46 (escitalopram group, days of escitalopram administration mean (SD) = 15.7 (2.70)). The study sample’s clinical and demographic information is summarised in Table 1, and a flowchart indicating participant enrolment and inclusion in data analysis compliant with the Consolidaed Standards for Reporting Trials (CONSORT) standards is shown in Supplemental Figure 13.

**Table 1.** Demographic description of the sample included in the study at baseline and after 2–3 weeks of escitalopram. *p*-values represent the difference between the placebo and drug groups at follow-up. Missing values were omitted from the comparisons (baseline BDI: 3 participants, baseline GAD-7, PHQ-9, STAI: 1 participant; follow-up BDI: 2 participants, baseline GAD-7, PHQ-9, STAI: 1 participant).

	Baseline		Follow-up				*p*
Measure	*n* = 96		Escitalopram (*n* = 46)		Placebo (*n* = 40)		
% Female	70.8		73.9		72.5		
	Mean	SD	Mean	SD	Mean	SD	*p*
Age	24.2	7.2	22.9	6.6	25.2	7.5	0.14
BDI	2	2.8	3.4	4.6	3.4	3.9	0.95
GAD-7	1.8	2.1	3.2	4.4	2.6	2.6	0.44
PHQ-9	1.8	3.2	3.4	4.0	2.1	2.0	0.07
STAI-S	29.8	7.4	32.9	11.2	32.2	7.9	0.73
STAI-T	33.2	7.7	33.6	8.0	34.2	7.4	0.71

BDI: the Beck depression inventory; GAD-7: generalized anxiety disorder 7; PHQ-9: the patient health questionnaire 9; STAI-S and STAI-T: the state-trait anxiety inventory (state and trait subscales); SD: standard deviation.

### Activation

#### Baseline

**Figure 2.**
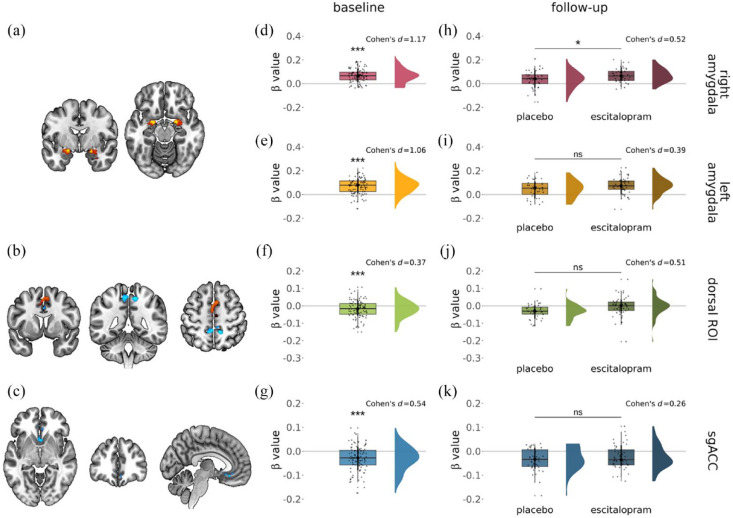
Activation changes during emotional face processing (faces vs fixation cross: red-yellow colour indicates increases, blue decreases) were seen within the (a) bilateral amygdala, (b) dorsal ROI and (c) sgACC. These baseline changes were recapitulated in the betas extracted from (d) the right amygdala, (e) the left amygdala, (f) dorsal ROI and (g) the sgACC. Significantly increased activation of the task was seen (h) in the right amygdala after subchronic escitalopram administration, but not within the (i) left amygdala, (j) dorsal ROI (although there was a trend level increase) and (k) sgACC. Significant results were considered at pSVC < 0.05 (small volume-corrected analyses) and *p* < 0.05 (average parameter analyses).

Consistent with our prior work, we found significant activation in all regions of interest at baseline (one-sample *t*-test, right amygdala: increased activation, *p* < 0.001, Cohen’s *d* = 1.17, left amygdala: increased activation, *p* < 0.001, Cohen’s *d* = 1.06, dorsal ROI: reduced activation, *p* ⩽ 0.001, Cohen’s *d* = 0.37, sgACC: reduced activation, *p* < 0.001, Cohen’s *d* = 0.54) (Figure 2(d)–(g)) as well as the control region (rFFA: increased activation, *p* < 0.001, Cohen’s *d* = 1.55) (Supplemental Figure 2(b)). The results obtained with average regional activation parameters were corroborated by the small volume-corrected analyses in AFNI for the right and left amygdala (Figure 2(a)), dorsal ROI (Figure 2(b)), the sgACC (Figure 2(c)) and the rFFA (Supplemental Figure 2(a)).

#### Follow-up treatment effects

The average activation parameters extracted from the regions from the follow-up scans were significantly higher in the escitalopram than in the placebo study group in the right amygdala (linear regression model adjusting for age, sex and baseline activation parameters, *p* = 0.034, Cohen’s *d* = 0.52) (Figure 2(h)). There were no significant group differences in the left amygdala (*p* = 0.13, Cohen’s *d* = 0.39) (Figure 2(i)), the dorsal ROI (*p* = 0.080, Cohen’s *d* = 0.51) (Figure 12(j)), the sgACC (*p* = 0.21, Cohen’s *d* = 0.26) (Figure 2(k)) or rFFA (*p* = 0.19, Cohen’s *d* = 0.33) (Supplemental Figure 2(c)). These differences were not found with the small volume correction analyses in AFNI (see Supplement).

### Connectivity

#### Baseline

**Figure 3.**
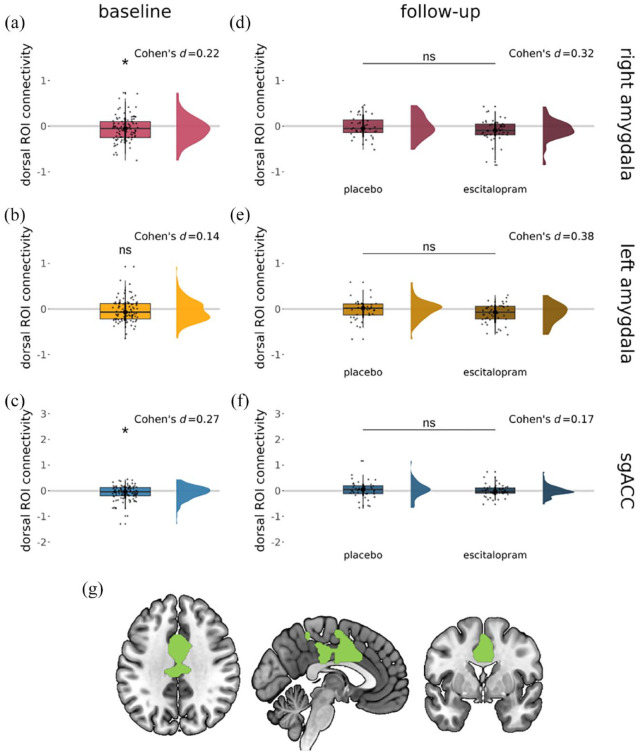
Significant connectivity (gPPI β value) during emotional face processing (faces vs fixation cross) between the dorsal ROI and (a) the right amygdala (but not (b) the left amygdala) and (c) the sgACC. No significant changes in connectivity between the dorsal ROI and the (d) right amygdala, (e) left amygdala or (f) sgACC were seen following subchronic escitalopram administration. (g) The dorsal ROI mask was used for these analyses. Significant results were considered at pSVC < 0.05 (small volume-corrected analyses) and *p* < 0.05 (average parameter analyses).

At baseline, there was significant connectivity during emotional face processing between the dorsal ROI and the right amygdala (one-sample *t*-test, *p* = 0.030, Cohen’s *d* = 0.22) and between the dorsal ROI and the sgACC (one-sample *t*-test, *p* = 0.011, Cohen’s *d* = 0.27) (Figure 3(a) and (c)), but not between the dorsal ROI and the left amygdala (one-sample *t*-test, *p* = 0.16, Cohen’s *d* = 0.14) (Figure 3(b)), when using average connectivity parameters. Complementary small volume-corrected analyses in AFNI corroborated significant negative connectivity between the dorsal ROI and the sgACC only (see Supplement).

#### Follow-up

There were no differences between groups at follow-up in the average connectivity parameter extracted from the dorsal ROI for either seed region (linear regression, right amygdala: *p* = 0.209, Cohen’s *d* = 0.32, left amygdala: *p* = 0.0731, Cohen’s *d* = 0.38, sgACC: *p* = 0.3820, Cohen’s *d* = 0.17) (Figure 3(d)–(f)) when including age, sex and baseline connectivity parameters as covariates of no interest. Similarly, equivalent analyses in AFNI did not reveal significantly altered connectivity at follow-up between the escitalopram and placebo groups for the dorsal ROI and either seed region (right amygdala, left amygdala or the sgACC).

## Discussion

Our primary finding is that 2- to 3-week escitalopram administration in healthy volunteers was associated with an increase in right amygdala activation during emotional face processing. By contrast, the left amygdala, dorsal ROI, sgACC and the rFFA showed no change in activation following the escitalopram administration, despite being robustly activated by the task. Similarly, replicated connectivity patterns at baseline were not modulated by escitalopram.

Our findings challenge the view that SSRI-mediated neurobiological changes are associated with a sustained reduction in amygdala activation. Some previous studies administering (es)citalopram acutely (i.e., as a single dose) found a decrease in amygdala activation during emotion processing, but other studies using such paradigm found an increase or no change in amygdala activation (decrease: Del-Ben et al., 2005; Grady et al., 2013; Harmer et al., 2006; Murphy et al., 2009; Windischberger et al., 2010; increase: Bigos et al., 2008; Selvaraj et al., 2017; no change: Brühl et al., 2009; Hornboll et al., 2018; Klomp et al., 2013). Moreover, findings of studies administering escitalopram or citalopram for a period sufficient for the drug to achieve a steady state (7–10 days) have been inconsistent (Arce et al., 2008; Harmer et al., 2006; Henry et al., 2013; Maron et al., 2016; McCabe et al., 2010; Norbury et al., 2009; Windischberger et al., 2010). This may have been due to the relatively low sample size in these studies (maximum of 16 participants per group), yielding low power. In the current study, we used a large group of healthy volunteers and saw an elevation of right amygdala activation after 2–3 weeks of escitalopram intake. This finding may represent a few potential mechanisms of SSRI action which we now discuss in turn.

There is evidence that serotonergic function in the amygdala is time-dependent. In rodents, acute SSRI administration was found to increase serotonin release into the amygdala (Bocchio et al., 2016) and to inhibit the firing rate of the serotonergic dorsal raphe nucleus (DRN) innervating it (Burghardt and Bauer, 2013). By contrast, 2- to 3-week SSRI administration was found to normalise DRN firing rates in the same animals (Burghardt and Bauer, 2013). This process may be associated with adaptive changes to the expression levels of the presynaptic 5-HT1A autoreceptors on DRN neurons, which may initially reduce their firing during temporarily increased synaptic serotonin levels (Commons, 2020; Sargin et al., 2019), but expression of which is then downregulated with long-term SSRI administration (Burghardt and Bauer, 2013; Di Giovanni et al., 2020). Moreover, acute SSRI administration in rodents was found to increase anxiety-related behaviour, whereas chronic administration was reported to reduce it (Bocchio et al., 2016; Burghardt et al., 2004; Ravinder et al., 2013), mirroring findings in human psychophysiological research (Grillon et al., 2007, 2009). Nevertheless, the neurobiological mechanisms of this apparent reversal and its time scale are unknown. On the one hand, evidence suggests an average of 4 weeks of treatment is required to alter mood in clinical populations (Jakubovski et al., 2019; Mendez et al., 2024). This would imply that our 2- to 3-week administration might be associated with effects similar to acute SSRI intake or fall at the mid-point of the reversal process, and if we had employed a longer administration period, we would have eventually seen reduced amygdala activation. This would be consistent with the clinically recognised phenomenon of an increase in subjective anxiety in the initial weeks of SSRI administration (Burghardt et al., 2004; Gollan et al., 2012; National Institute for Health and Care Excellence, 2020; Sinclair et al., 2009; Westenberg and Den Boer, 1989). As amygdala hyperactivation is a robust finding in anxiety disorders (Kim et al., 2011; Kolesar et al., 2019; Pantazatos et al., 2013), it is plausible that this initial anxiogenesis is a result of increases in anxiety-related brain activation (Chavanne and Robinson, 2021; Fox and Shackman, 2019). However, it has been suggested that symptom reduction may start in the second week of antidepressant treatment (Baldwin et al., 2014; Katzman et al., 2014), which is equivalent to the mean time of antidepressant treatment in our ITT sample. Consistently with this, we did not find an elevation in subjective anxiety ratings. While this may be due to the healthy sample not being vulnerable to increased amygdala activation, it is also plausible that changes in amygdala activation associated with antidepressant administration are not directly indicative of symptom reduction.

Another potential explanation for increased amygdala activation is that the effects of serotonin reuptake inhibition on amygdala activation during emotional face processing are dependent on the baseline state of the person’s serotonergic system. Looking at other neurotransmitter systems, it has been shown that pharmacologically increasing dopaminergic function improves cognitive function in individuals with lower baseline dopamine levels, whereas it can impair cognitive task performance in people with higher baseline dopaminergic function (Cools and D’Esposito, 2011). If this pattern is similar for serotonin neurotransmission, the increased amygdala activation we observed could be selective for our healthy sample, who may putatively have higher baseline serotonergic levels. In that case, the increase in amygdala activation could reflect changes away from homeostasis in healthy individuals, rather than its reinstatement as in pathological anxiety. To clarify this, future work should explore baseline serotonergic function and its modulation by SSRIs in patient samples.

It should be noted that our finding of elevated right amygdala activation during emotional face processing is in contrast to the results of three previous fMRI studies with subchronic (2–3 weeks) escitalopram administration, which found no differences in amygdala activation to the task (Arce et al., 2008; Henry et al., 2013; Simmons et al., 2009). Additionally to the sample size considerations mentioned earlier, it should be noted that all three of these studies used a within-subject design. We previously found that there was significant variability in the measurement of amygdala activation during emotional face processing within the same individuals scanned 2 weeks apart (Nord et al., 2017). This may indicate that the previous studies employing subchronic escitalopram administration might not have detected any changes in amygdala activation due to instability of the task fMRI BOLD signal, a low sample size or both, rather than providing evidence for a true lack of effect.

It is also important to note that our findings are in the context of a successful replication of the task activation and connectivity pattern. Previously, we found robust activation during emotion processing in the bilateral amygdala, dorsal ROI and rFFA, concomitant with reduced activation in the sgACC (Nord et al., 2017). We also found a task-specific increase in the connectivity of the dorsal ROI with the bilateral amygdala, but not with the sgACC (Nord et al., 2019). The former result also replicates other studies, which also found task-specific increases in amygdala connectivity with the dorsomedial prefrontal cortex during emotion-related paradigms (Gold et al., 2015; Vytal et al., 2014), albeit not all (Outhred et al., 2015). In the present study, using the same paradigm but a markedly larger sample, we replicated the pattern of activation to the task. This result is also concordant with the literature, showing robust activation in the amygdala and prefrontal cortices during emotion processing (Fusar-Poli et al., 2009; Phillips et al., 2003). Moreover, we replicated our previous finding of significant task-specific connectivity between the dorsal ROI and the right, but not the left amygdala. Additionally, we found significant task-specific positive connectivity between the dorsal ROI and the sgACC which was not seen in our prior paper (Nord et al., 2019). In our previous studies, we found that while amygdala connectivity with the dorsal ROI generated a signal of reliable magnitude across runs 2 weeks apart, its activation did not (Nord et al., 2017, 2019). This may suggest that the pattern of brain activation to emotional face-processing tasks is robust and present in independent samples, whereas brain connectivity during such tasks is not as reliable.

Interestingly, we did not find evidence suggesting escitalopram modulates either bilateral amygdala or sgACC connectivity with the dorsal ROI. This result is less easily interpretable as to our knowledge, there has only been one study investigating SSRI-induced changes in brain connectivity during an emotion-processing task with the gPPI approach, which did not use any of our regions of interest (Reed et al., 2022). However, we may have expected a modulation of connectivity between regions comprising the brain circuit underpinning anxiety expression. Previous studies in anxiety disorders have found inconsistent results, some showing increases, some reductions and some unchanged amygdala connectivity with the dorsal or dorsomedial prefrontal cortex (increases: Reinecke et al., 2015; Robinson et al., 2014; reductions: Kaldewaij et al., 2019; unchanged: Böhnlein et al., 2021; Duval et al., 2020). Similarly, no alterations in dmPFC or vmPFC connectivity with the amygdala were found in anxiety disorders (dmPFC: Cui et al., 2022; Duval et al., 2020; vmPFC: De la Peña-Arteaga et al., 2022). Therefore, the reason for the lack of SSRI-induced modulation of brain connectivity in our study between the regions of interest remains unclear. It is plausible that it reflected no modulation of the brain network underlying anxiety. Alternatively, it might have been related to the lack of subjective anxiety change in our sample. Finally, it is possible that the temporal timelines of changes in brain activation and connectivity during SSRI administration are different. More research is needed to elucidate this question.

A few methodological considerations of this study are of note. Firstly, we performed complementary analyses using extracted average activation and connectivity parameters and using cluster inference with small volume correction in AFNI. The elevation in right amygdala activation we found was only seen with the former approach. The findings may indicate that this increase is distributed across the right amygdala rather than localised to a specific sub-region. Further investigations with methods with increased resolution, such as 7T fMRI scanning, may help elucidate this incongruity. It is also noteworthy that we ascertained compliance by self-report but not with additional measures such as blood testing; therefore, some non-adherence may have been present within our sample. Additionally, the results we present were acquired from a sample of healthy volunteers, so our findings may not generalise to patient populations. Finally, as our paradigm included both emotional and neutral faces, the significance of our results for a particular aspect of emotional face processing (e.g. valence or salience processing) cannot be ascertained. However, not only is the paradigm we used a robust design employed by many previous studies (Fusar-Poli et al., 2009), but crucially, it was the same one used by the study we aimed to replicate (Nord et al., 2017, 2019). As such, future studies using more individual facial emotions, more trials per valence and/or a randomised order of stimuli eliciting more individual haemodynamic responses are warranted.

In conclusion, we found evidence that escitalopram treatment of just over 2 weeks in healthy volunteers may selectively increase right amygdala activation during emotional face processing, but not its connectivity with the dorsomedial cortex. Future research over longer periods and in patient populations will help elucidate the time scale and generalisability of these results. Regardless of the exact mechanisms that our findings represent, this is the most well-powered study of an SSRI in a healthy control sample to date and it indicates that, at least in healthy controls, the simple story of antidepressants reducing amygdala activation when given over multiple weeks may not be correct.

## Supplemental Material

sj-docx-1-jop-10.1177_02698811241286773 – Supplemental material for Amygdala activity after subchronic escitalopram administration in healthy volunteers: A pharmaco-functional magnetic resonance imaging study Supplemental material, sj-docx-1-jop-10.1177_02698811241286773 for Amygdala activity after subchronic escitalopram administration in healthy volunteers: A pharmaco-functional magnetic resonance imaging study by Paulina B Lukow, Millie Lowther, Alexandra C Pike, Yumeya Yamamori, Alice V Chavanne, Siobhan Gormley, Jessica Aylward, Tayla McCloud, Talya Goble, Julia Rodriguez-Sanchez, Ella W Tuominen, Sarah K Buehler, Peter Kirk and Oliver J Robinson in Journal of Psychopharmacology
