# Supplementary material for: Amygdala activity after subchronic escitalopram administration in healthy volunteers: A pharmaco-functional magnetic resonance imaging study
Source: J Psychopharmacol. 2024 Oct 4;38(12):1071–82. doi: 10.1177/02698811241286773 (PMC11531087; doi:10.1177/02698811241286773)
Supplement: sj-docx-1-jop-10.1177_02698811241286773 – Supplemental material for Amygdala activity after subchronic escitalopram administration in healthy volunteers: A pharmaco-functional magnetic resonance imaging study [file sj-docx-1-jop-10.1177_02698811241286773.docx]

**Supplemental Material**

***ROI masks***


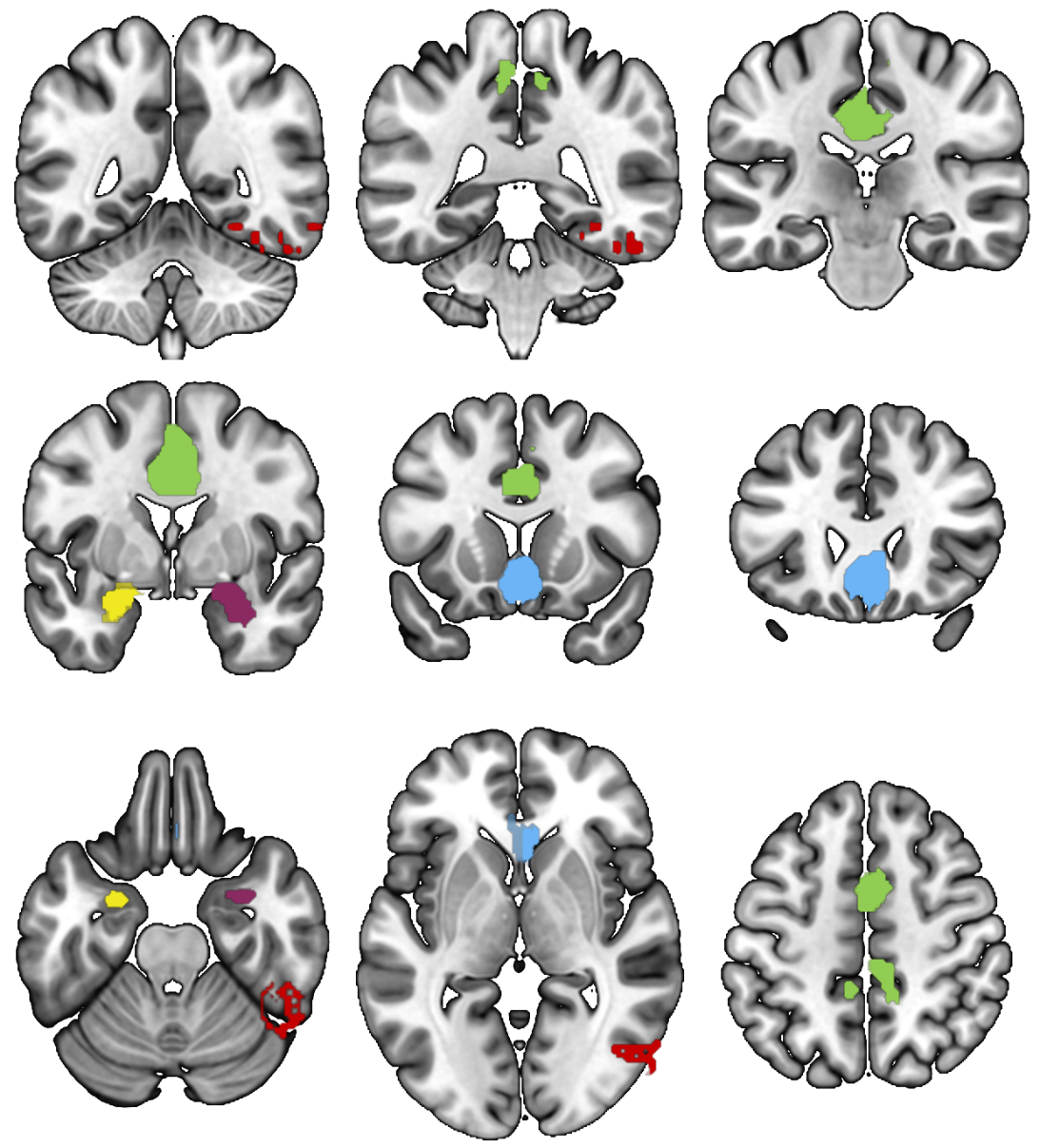


**Supplementary Figure 1.** Masks used for the region of interest analyses. Red, right fusiform face area; green, dorsal ROI; yellow, left amygdala; purple, right amygdala; blue, subgenual anterior cingulate cortex.

***Activation and connectivity analyses in AFNI***

Analyses equivalent to those performed with the extracted average activation and connectivity parameters were conducted in AFNI with its *3dttest++* with the integrated -Clustsim option, which estimates minimum cluster size and calculates significance of each cluster resulting from an analysis. To investigate baseline brain activation and connectivity for the contrast of interest (faces vs fixation cross), a two-tailed one-sample t-test was performed on all beta-weight activation maps passing the quality assessment, resulting from within-subject modelling described above. Participant sex and age were entered into the model as covariates of no interest. The assumed cluster-defining threshold was set to voxel-wise p_uncorrected_<0.001, and the final results were considered significant if passing a corrected threshold of p_cluster_<0.05.

To investigate the potential difference in brain activation or connectivity for the contrast of interest at follow-up between the placebo and escitalopram study groups, a two-tailed two-sample t-test was performed on all available follow-up beta-weight activation maps passing the quality assessment, resulting from within-subject modelling described above. Participant sex, age and voxel-wise brain activation at baseline were entered into the model as covariates of no interest. This was to avoid the assumption that the study groups may have differed at baseline, a practise that was suggested not to be justified if an appropriate randomisation method was followed [83–85], as was the case in our study. The assumed cluster-defining threshold was set to voxel-wise p_uncorrected_<0.001, and the final results were considered significant if passing a corrected threshold of p_cluster_<0.05.

***Baseline activation analysis in AFNI***

In small volume correction analyses in AFNI, there was bilateral amygdala activation at baseline (right: k=184, xyz=22,-5,-17, p<0.01 and k=4, xyz=32,-5,-29, p<0.03; left: k=152, xyz=-25,-3,-15, p<0.01) (Figure 1A) during emotion processing. There were both clusters of elevated (k=260, xyz=-3,6,52, p<0.01) and reduced (k=734, xyz=14,-45,56, p<0.01; k=76, xyz=-3,6,52, p<0.01; k=16, xyz=4,-17,56, p<0.02) activation in the dorsal ROI (Figure 1B). Participants showed reduced activation to the task in the subgenual cingulate cortex, accordingly with our hypotheses (k=171, xyz=-5,40,-5, p<0.01; k=36, xyz=6,40,-11, p<0.01) (Figure 1C). In the positive control region, the right fusiform area, there were two clusters of increased activation (k=299, xyz=44,-59,-25, p<0.01 and k=136, xyz=52,-75,-5, p<0.01) and one small cluster of reduced activation to the task (k=14, xyz=34,-41,-13, p<0.01) (Supplementary Figure 2A).


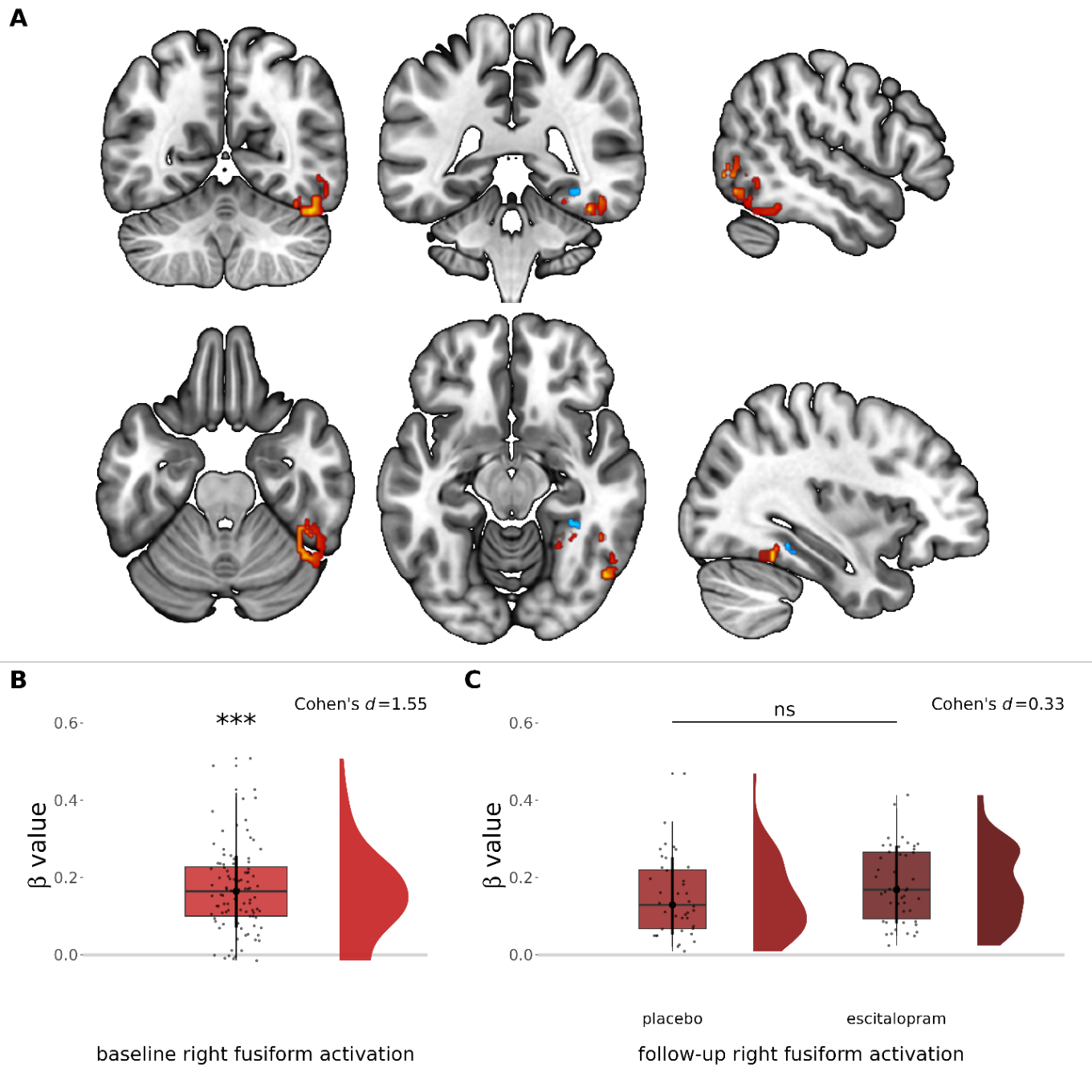


**Supplementary Figure 2.** Activation in the control region, the right fusiform area, to the emotion processing task (faces vs fixation cross). (**A**) Clusters of increased activation to the task in the right fusiform area activation to the task at baseline. (**B**) Baseline activation parameter extracted from the right fusiform area. (**C**) Comparison of activation parameters between the placebo and escitalopram groups at follow-up in the right fusiform area.

***Baseline connectivity analysis in AFNI***

Complementary analyses in AFNI corroborated significant negative connectivity between the dorsal ROI and the sgACC (k=10, xyz=-3,-19,30, p<0.03) (Supplementary Figure 3) but not the right or left amygdala.


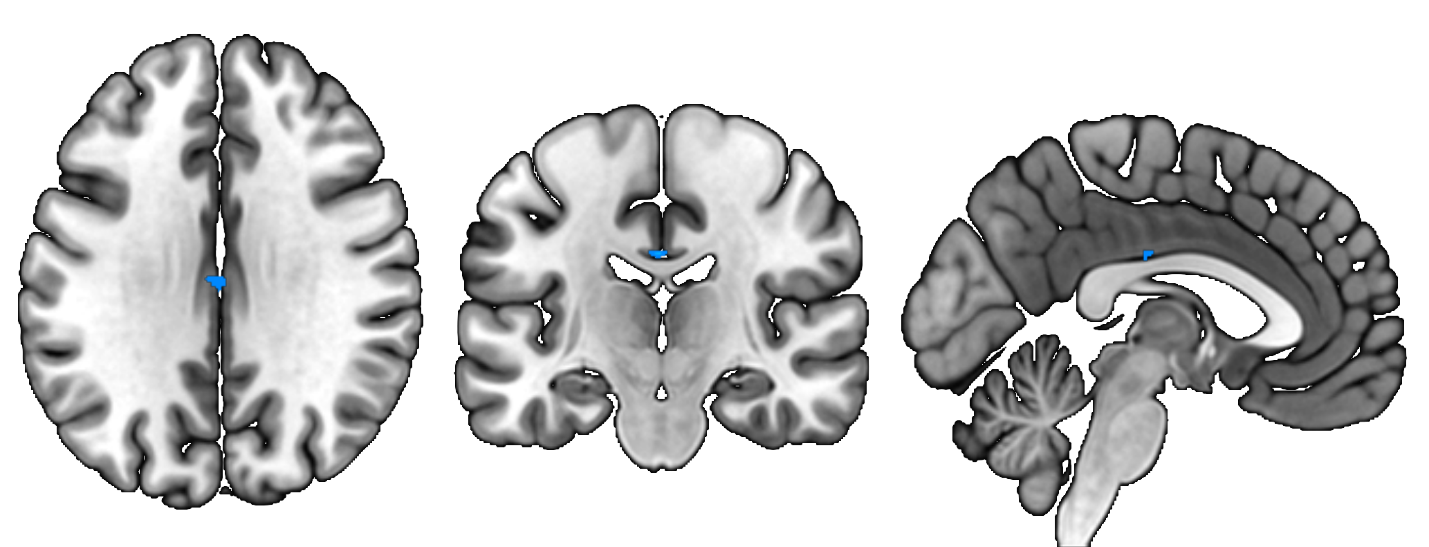


**Supplementary Figure 3.** Significant negative connectivity between the dorsomedial region of interest and the subgenual anterior cingulate cortex.

***Follow-up analyses within the dorsomedial cortical clusters of significant baseline activation***

We sought to further investigate whether the lack of statistically significant difference in activation parameters between the placebo and escitalopram study groups in the dorsal ROI at follow-up was due to the varied activation patterns within this region, as suggested by both clusters of increased and reduced activation to the task at baseline. To this end, we generated masks based on the four activation clusters found in the baseline analysis (Figure 2B) and investigated differences in activation within these masks (Supplementary Figure 4) as well as in connectivity (Supplementary Figures 5-7) at follow-up.

***Activation***

The lack of statistically significant difference in activation parameters between the placebo and escitalopram study groups in the dorsal ROI was replicated for all clusters in this region found to show reduced activation to the task at baseline (Supplementary Figure 4A, C, D), while the cluster found to show elevated activation at baseline narrowly avoided significance (Supplementary Figure 4B).

***Connectivity***

The lack of statistically significant difference in connectivity with the dorsal ROI region between the placebo and escitalopram study groups was replicated for all clusters in this region found to show significantly elevated or reduced activation to the task at baseline (Supplementary Figures 5-7). However, it is noteworthy that two of the comparisons narrowly avoided significance: one of the connectivity between the second largest cluster of hypoactivation to the task within the dorsal ROI and the right amygdala (Supplementary Figure 5C), and one of the connectivity between the largest cluster of hypoactivation to the task within the dorsal ROI and the left amygdala (Supplementary Figure 6A).


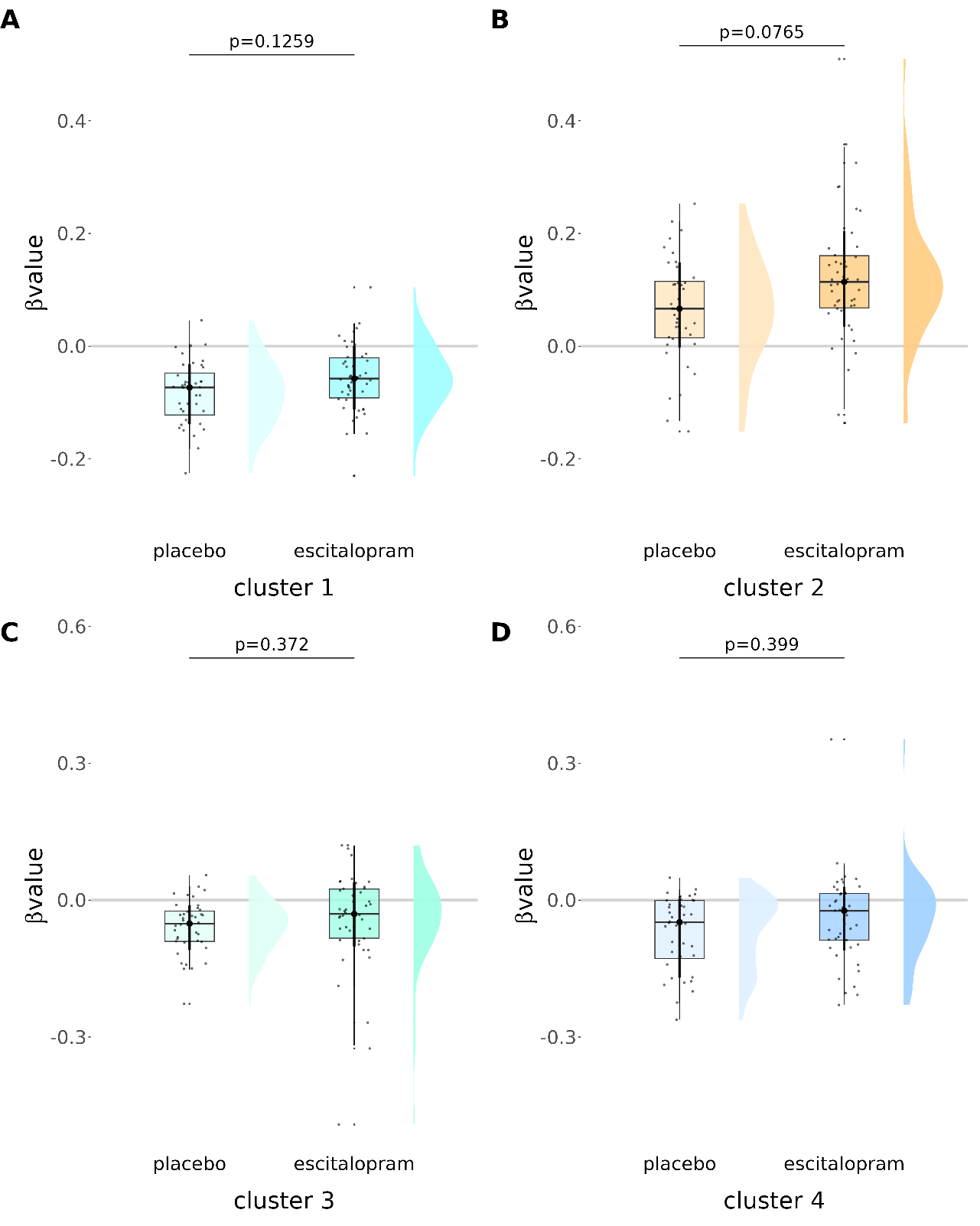


**Supplementary Figure 4.** A lack of statistically significant difference in activation parameters between the placebo and escitalopram study groups at follow-up within the individual clusters of significant activation to the task at baseline. (**A, C, D**) Clusters found at baseline to show reduced activation to task, (**B**) cluster found at baseline to show increased activation to task. Clusters shown in descending size order **A-D**.

**
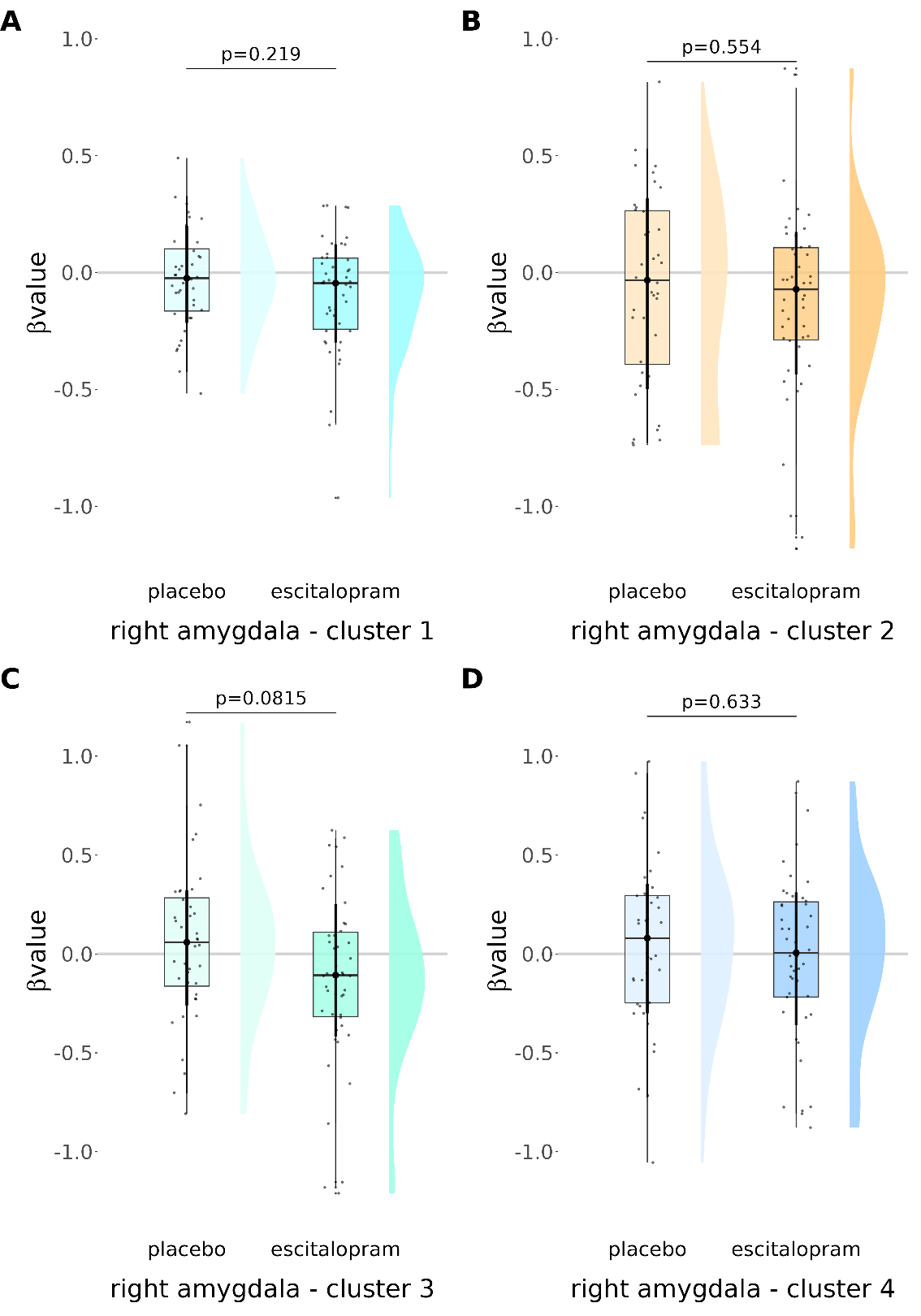
**

**Supplementary Figure 5.** A lack of statistically significant difference in connectivity parameters between the placebo and escitalopram study groups at follow-up, between the right amygdala and the individual clusters of significant activation to the task at baseline. (**A, C, D**) Clusters found at baseline to show reduced activation to task, (**B**) cluster found at baseline to show increased activation to task. Clusters shown in descending size order **A-D**.

**
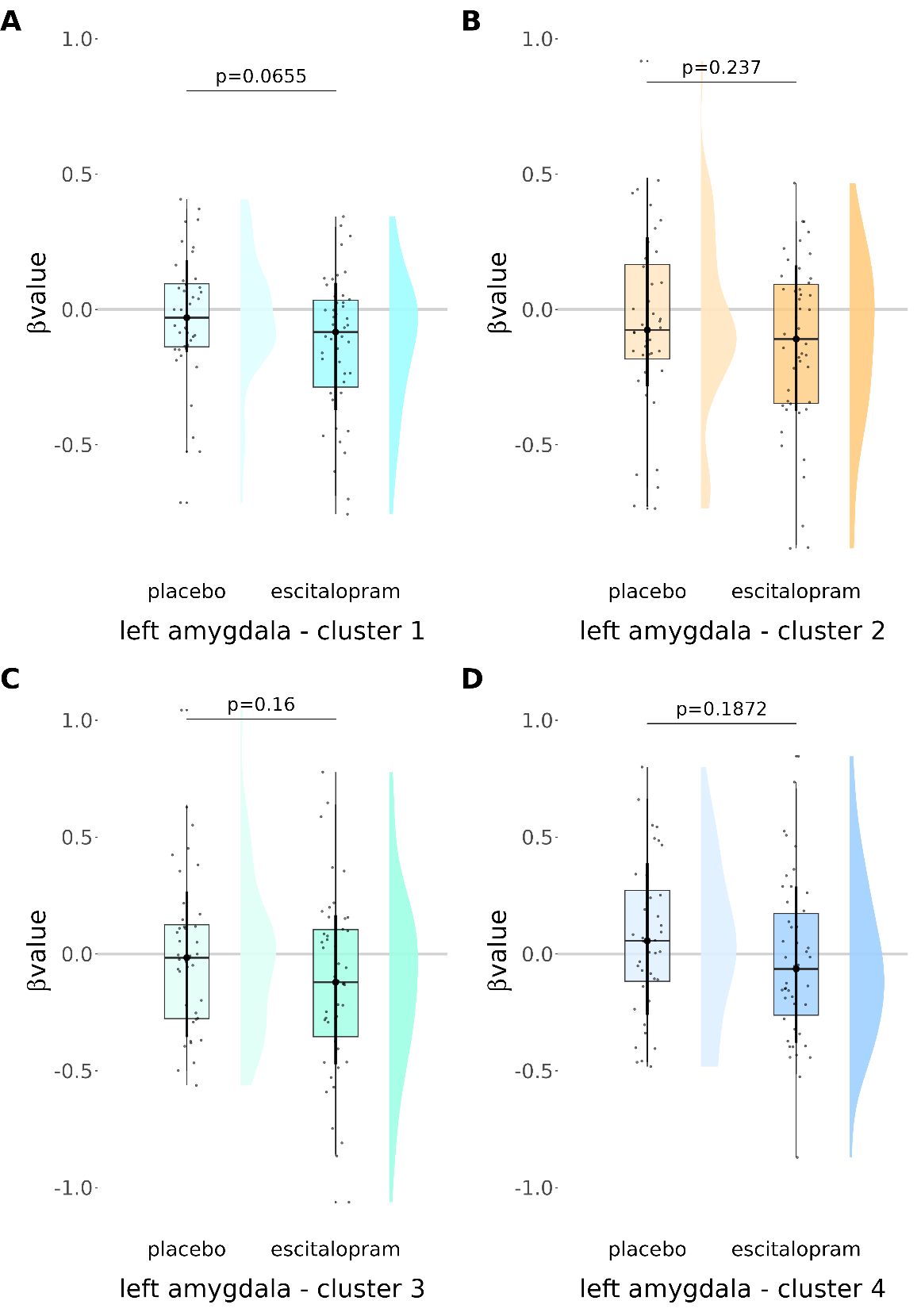
**

**Supplementary Figure 6.** A lack of statistically significant difference in connectivity parameters between the placebo and escitalopram study groups at follow-up, between the left amygdala and the individual clusters of significant activation to the task at baseline. (**A, C, D**) Clusters found at baseline to show reduced activation to task, (**B**) cluster found at baseline to show increased activation to task. Clusters shown in descending size order **A-D**.

**
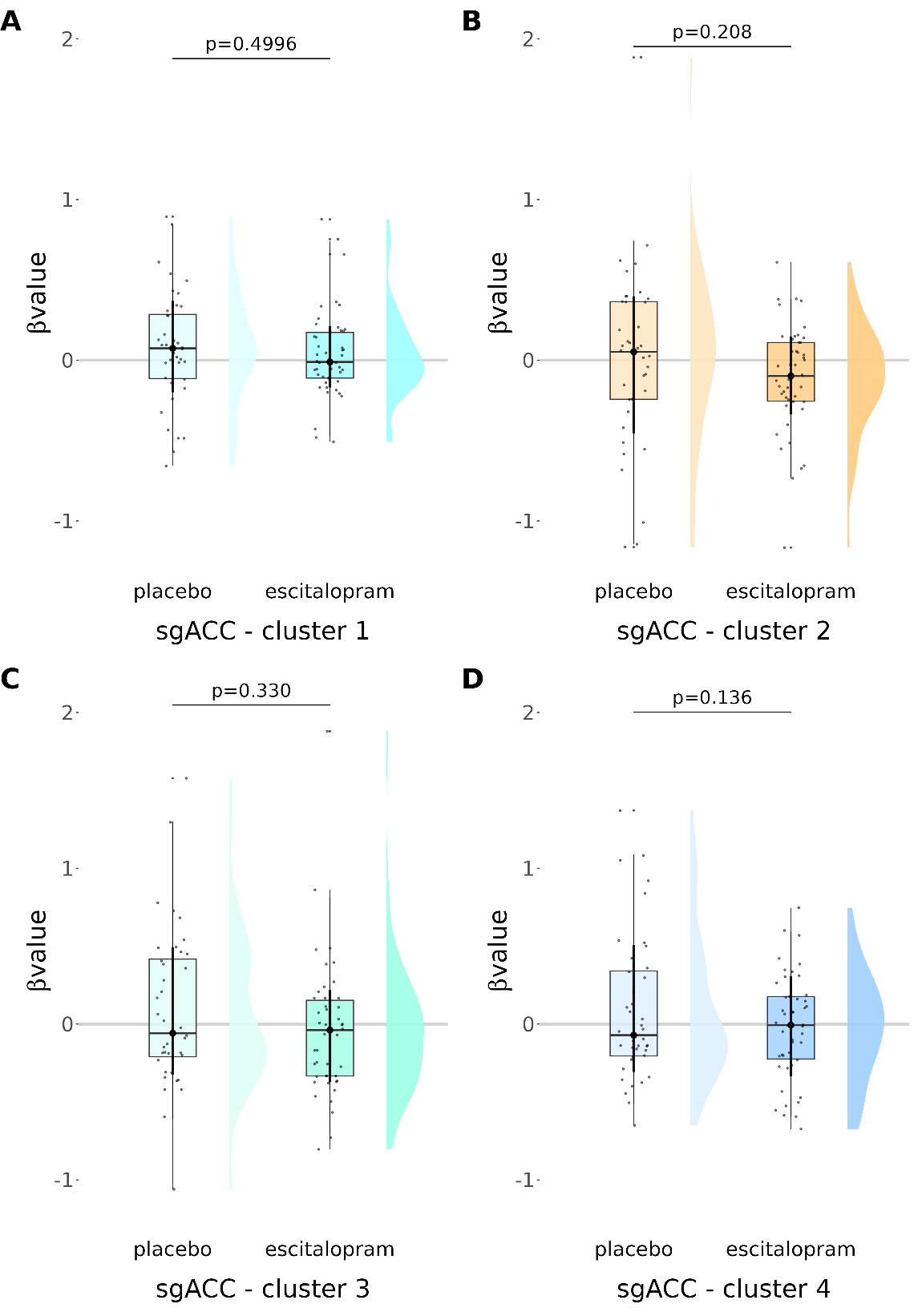
**

**Supplementary Figure 7.** A lack of statistically significant difference in connectivity parameters between the placebo and escitalopram study groups at follow-up, between the subgenual anterior cingulate cortex and the individual clusters of significant activation to the task at baseline. (**A, C, D**) Clusters found at baseline to show reduced activation to task, (**B**) cluster found at baseline to show increased activation to task. Clusters shown in descending size order **A-D**.

***Follow-up activation analyses with baseline data subtraction***

***Activation***

There were no differences between the placebo and escitalopram arms of the study in the comparison of images resulting from the subtraction of the baseline unthresholded activation maps from the follow-up unthresholded activation maps in either region tested. This was the case for the analyses performed in AFNI as well as those on extracted activation parameters in R (linear regression model with age and sex as covariates of no interest, right amygdala: p=0.436, left amygdala: p=0.852, dorsal ROI: p=0.502, sgACC: p=0.250, right fusiform: p=0.435) (Supplementary Figure 8).

***Connectivity***

At follow-up, there were no differences between the placebo and escitalopram arms of the study in the comparison of images resulting from the subtraction of the baseline unthresholded connectivity maps from the follow-up unthresholded connectivity maps. This was the case for the analyses performed in AFNI as well as those on extracted activation parameters in R (linear regression with age and sex as covariates of no interest, right amygdala: p=0.610, left amygdala: p=0.790, sgACC: p=0.5923) (Supplementary Figure 9).


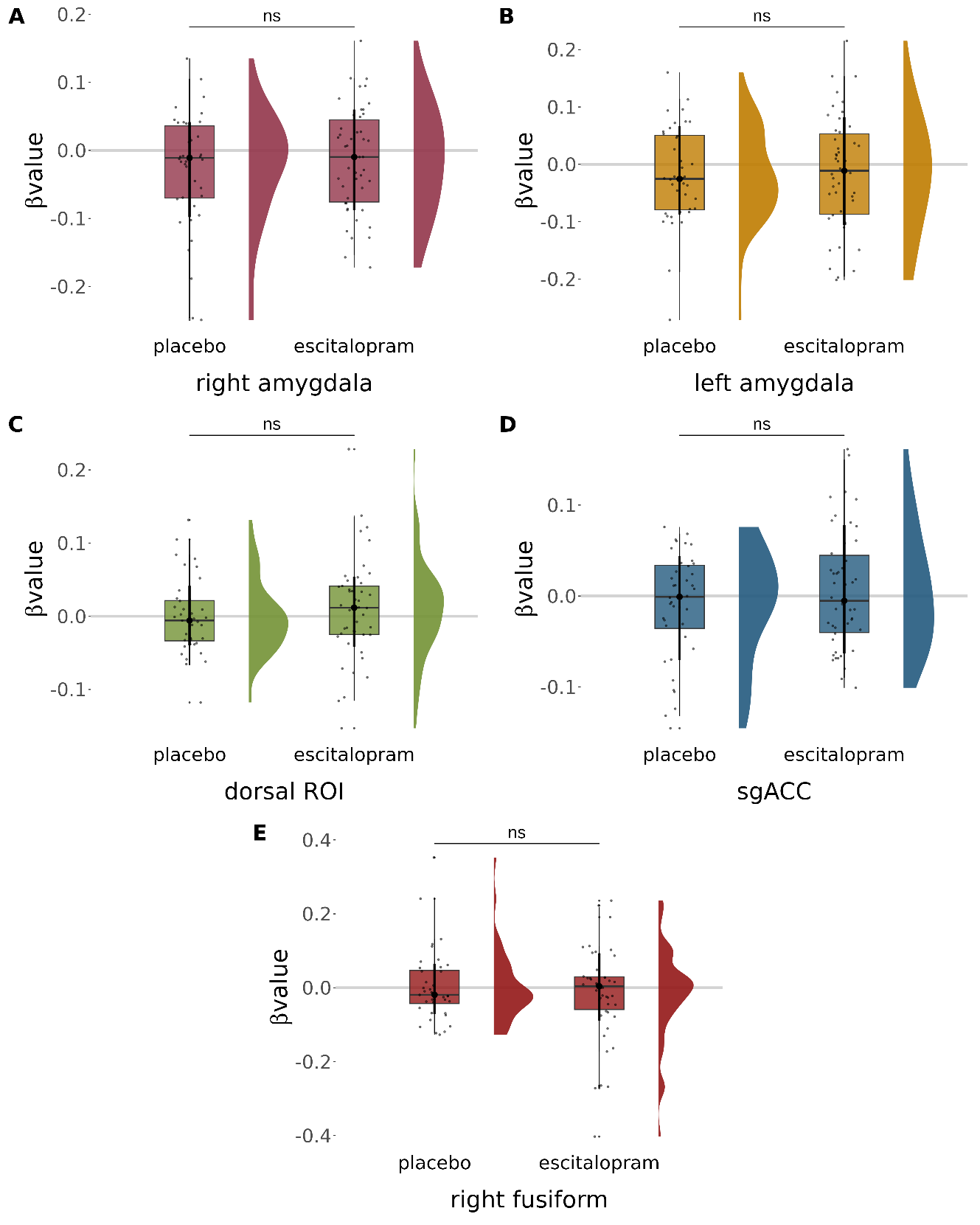


**Supplementary Figure 8.** Comparison of activation parameters extracted from images resulting from the subtraction of the baseline unthresholded activation maps from the follow-up unthresholded activation maps between the placebo and escitalopram groups in (**A**) the right amygdala, (**B**) the left amygdala, (**C**) the dorsal region of interest, (**D**) the subgenual anterior cingulate cortex and (**E**) the right fusiform area.

*
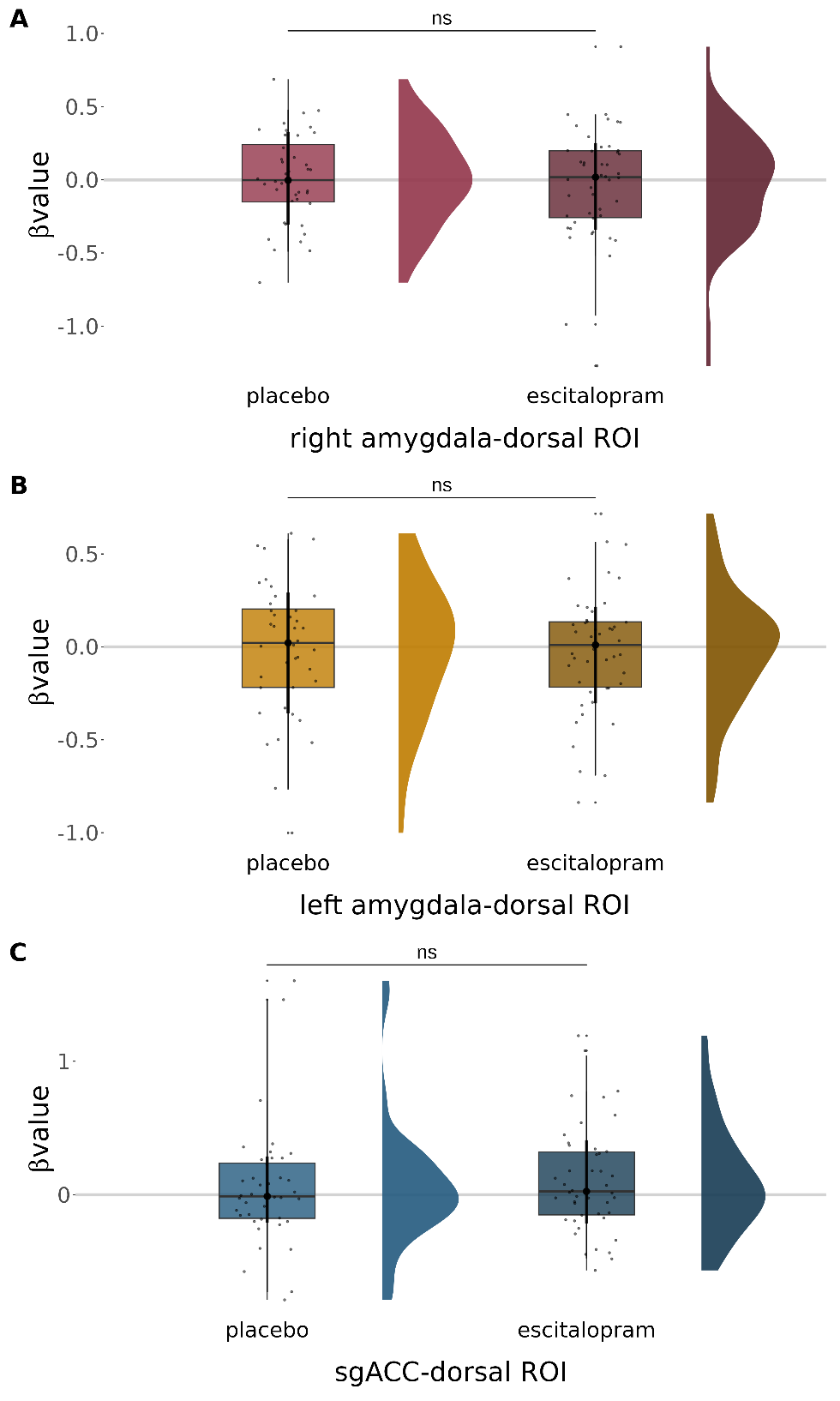
*

**Supplementary Figure 9.** Lack of significant difference in connectivity between the dorsal ROI and (A) right amygdala, (B) left amygdala and (C) subgenual anterior cingulate cortex, after subtraction of the baseline unthresholded beta-weight maps from the follow-up unthresholded beta-weight maps.

***Relationship of regional brain activation and connectivity with trait anxiety***

We also sought to investigate the relationship between trait anxiety and brain activation and connectivity during emotion processing. To this end, we performed regression analyses in AFNI and R, this time including trait anxiety scores as an additional regressor of interest. Trait anxiety included in all these analyses was measured with the State-Trait Anxiety Inventory (24) on the day of each scanning session. In AFNI, *3dttest++* with the -Clustsim option was used to perform one-sample and two-sample t-tests. The data entered into the two-sample t-test comprised the follow-up neuroimaging data. In R, linear regression models were estimated with the *lm* function using, first, baseline activation or connectivity parameters, and second, follow-up activation or connectivity parameters extracted from the regions of interest. In the follow-up analyses, we investigated the relationship between brain activation or connectivity and trait anxiety depending on study arm status using an interaction term in the linear regression model. All analyses included age and sex as covariates of no interest and were also repeated with the baseline activation or connectivity parameters as additional covariates of no interest.

***Activation***

At baseline, there was a small but significant cluster of association between trait anxiety and brain activation in the control region, the right fusiform area (k=4, xyz=50,-63,-5) (Supplementary Figure 10). There were no clusters of such association in the right amygdala, left amygdala, dorsal ROI or the sgACC. At follow-up, there were no clusters of such association depending on group status in either tested region.

There was an association between trait anxiety and brain activation parameter extracted from the right fusiform area at baseline (linear regression model, right fusiform area: p=0.0167) (Supplementary Figure 11E) but not from the other regions of interest (right amygdala: p=0.38, left amygdala: p=0.20, dorsal ROI: p=0.88, sgACC: p=0.75) (Supplementary Figure 11A-D). There was no association between trait anxiety and brain activation extracted from either region of interest the dorsal ROI (linear regression model adjusting for sex, age and baseline regional activation, group*trait anxiety interaction: (right amygdala (p=0.816), left amygdala (p=0.851), dorsalROI (p=0.1808), sgACC (p=0.7565) and the right fusiform area (p=0.824) depending on group status (Supplementary Figure 11F-J).

***Connectivity***

There were no clusters of significant association between trait anxiety and brain connectivity with the dorsal ROI in the left amygdala, right amygdala or the sgACC, at baseline or follow-up.

There were no associations between trait anxiety and parameters of brain connectivity with the dorsal ROI extracted from the areas of interest at baseline (linear regression model, right amygdala: p=0.55, left amygdala: p=0.60, sgACC: p=0.43) (Supplementary Figure 12A-C). At follow-up, there was an association between trait anxiety and parameter of connectivity with the dorsal ROI extracted from the right amygdala (linear regression mode with group*trait anxiety interaction, p=0.0497) (Supplementary Figure 12D), but not in the left amygdala (p=0.188) or the sgACC (p=0.1994) (Supplementary Figure 12E-F).


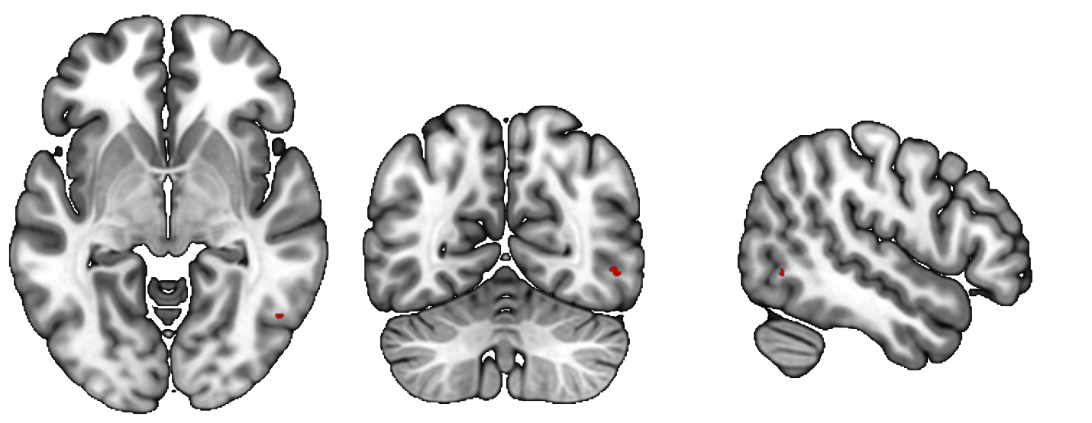


**Supplementary Figure 10.** Positive association between baseline trait anxiety and brain activation to the emotion processing task in the control region, the right fusiform area.

**
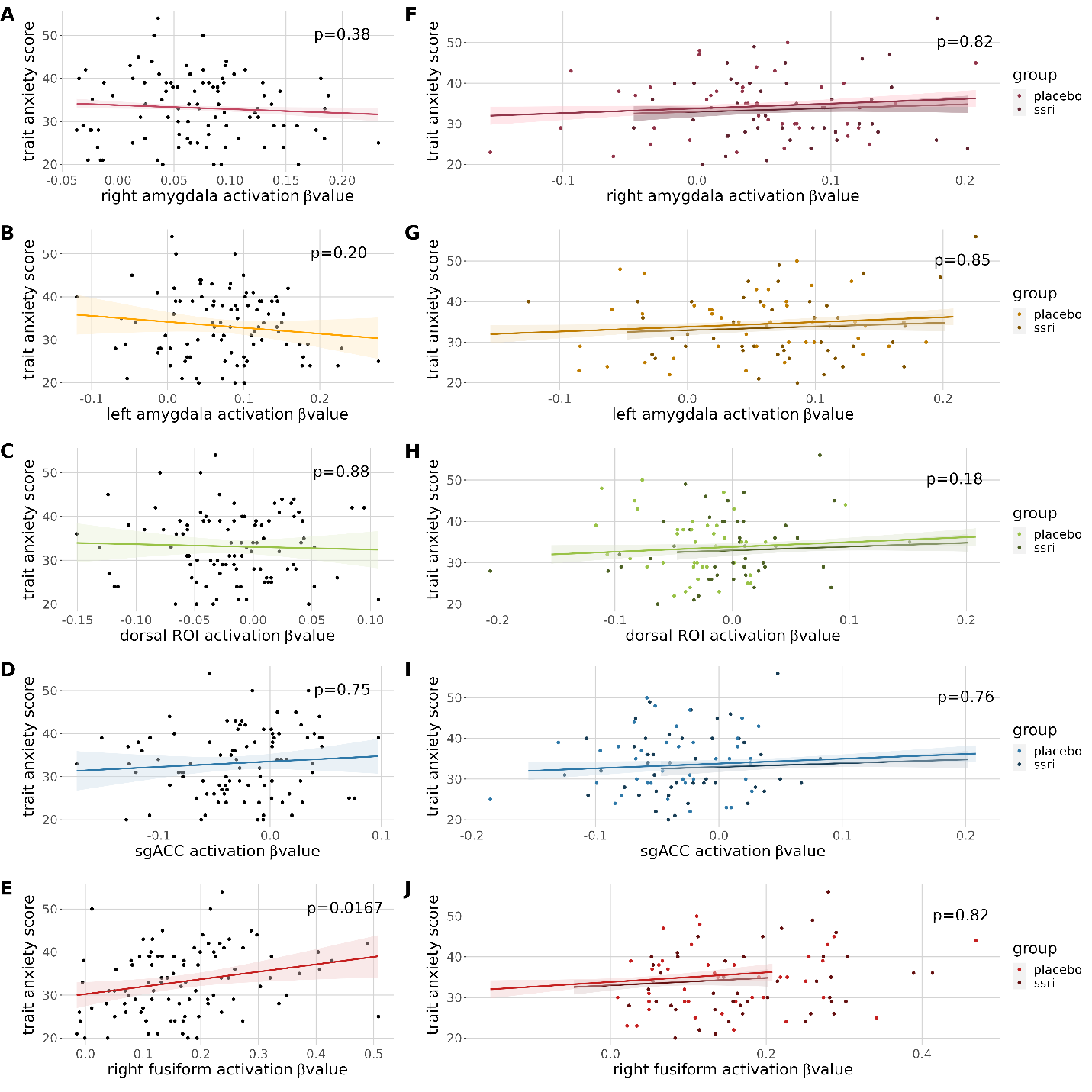
**

**Supplementary Figure 11.** Associations between trait anxiety and brain activation parameters extracted from the areas of interest at baseline (**A**-**E**) and at follow-up after 14-21 days of escitalopram or placebo treatment (**F**-**J**).

**
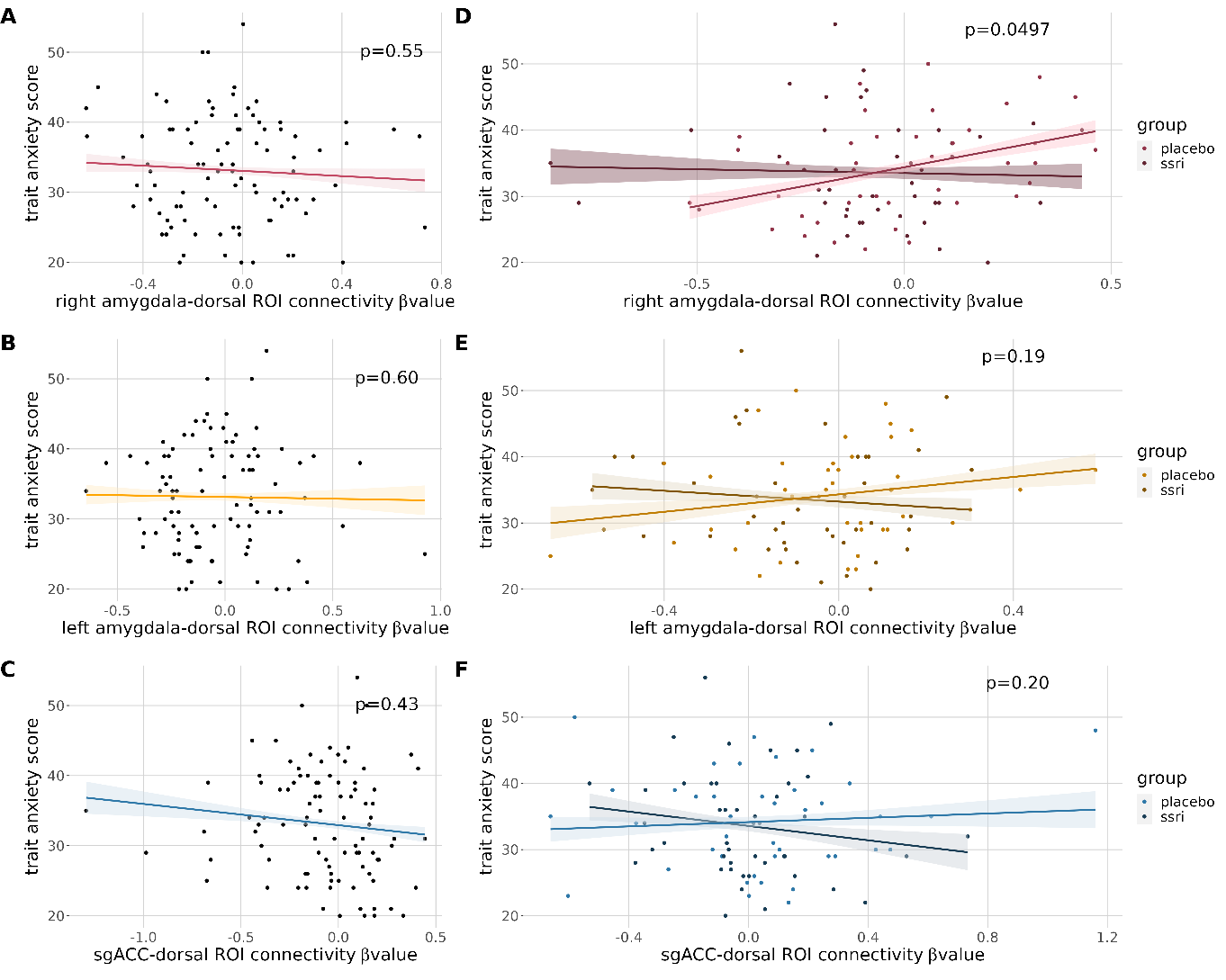
**

**Supplementary Figure 12.** Associations between trait anxiety and parameters of connectivity with the dorsal region of interest extracted from the areas of interest at baseline (**A**-**C**) and at follow-up after 14-21 days of escitalopram or placebo treatment (**D**-**F**).

***Medication side effects***

All study participants were asked to record any medication side effects experienced at days 3, 7 and 14 of the drug administration. The side effects included in the questionnaire were ‘Altered sleep pattern’, ‘Changes in mood (euphoric/depressed)’, Fatigue / (muscle) weakness’, ‘Restlessness’, ‘Dizziness’, ‘Changes in appetite’, ‘Nausea’, ‘Weight loss / gain’, ‘Excess sweating / dryness of skin’, ‘Palpitations’, ‘Infections / colds / …’ and ‘Other’. At all the time points, both escitalopram and placebo groups reported no side effects at least 80% of the time and severe side effects were reported at less than 1.5% of the time. There were no serious adverse events.

| Day | Severity | Medication group | Percentage of reports |
| --- | --- | --- | --- |
| 3 | Absent | escitalopram | 80.8 |
|  |  | placebo | 89.9 |
|  | Mild | escitalopram | 13 |
|  |  | placebo | 8.2 |
|  | Moderate | escitalopram | 4.9 |
|  |  | placebo | 1.9 |
|  | Severe | escitalopram | 1.3 |
|  |  | placebo | 0 |
| 7 | Absent | escitalopram | 82.7 |
|  |  | placebo | 88.9 |
|  | Mild | escitalopram | 12.7 |
|  |  | placebo | 8.2 |
|  | Mild/moderate | escitalopram | 0.2 |
|  |  | placebo | 0 |
|  | Moderate | escitalopram | 4 |
|  |  | placebo | 2.5 |
|  | Severe | escitalopram | 0.4 |
|  |  | placebo | 0.4 |
| 14 | Absent | escitalopram | 85.4 |
|  |  | placebo | 90.6 |
|  | Mild | escitalopram | 12 |
|  |  | placebo | 7.6 |
|  | Moderate | escitalopram | 2 |
|  |  | placebo | 1.8 |
|  | Severe | escitalopram | 0.6 |
|  |  | placebo | 0 |

**Supplementary Table 1.** Frequency of side effects reported by participants of this study at day 3, 7 and 14 of escitalopram or placebo administration. Data includes all available ratings for all participants enrolled on the study.

**Supplementary Figure 13.** A flowchart indicating participant enrolment and inclusion in data analysis, compliant with the CONSORT standards.

**
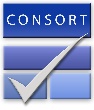
CONSORT 2010 checklist of information to include when reporting a randomised trial***

| **Section/Topic** | **Item No** | **Checklist item** | **Reported on page No** |
| --- | --- | --- | --- |
| **Title and abstract** | | | |
|  | 1a | Identification as a randomised trial in the title | Not applicable |
|  | 1b | Structured summary of trial design, methods, results, and conclusions (for specific guidance see CONSORT for abstracts) | Not applicable |
| **Introduction** | | | |
| Background and objectives | 2a | Scientific background and explanation of rationale | 4-6 |
|  | 2b | Specific objectives or hypotheses | 6 |
| **Methods** | | | |
| Trial design | 3a | Description of trial design (such as parallel, factorial) including allocation ratio | 7-8 and Supplemental Material p. 18 |
|  | 3b | Important changes to methods after trial commencement (such as eligibility criteria), with reasons | Not applicable |
| Participants | 4a | Eligibility criteria for participants | 7 |
|  | 4b | Settings and locations where the data were collected | 7 and 10 |
| Interventions | 5 | The interventions for each group with sufficient details to allow replication, including how and when they were actually administered | 8 |
| Outcomes | 6a | Completely defined pre-specified primary and secondary outcome measures, including how and when they were assessed | 8, 9, 11 |
|  | 6b | Any changes to trial outcomes after the trial commenced, with reasons | Not applicable |
| Sample size | 7a | How sample size was determined | 3 |
|  | 7b | When applicable, explanation of any interim analyses and stopping guidelines | Not applicable |
| Randomisation: |  |  |  |
| Sequence generation | 8a | Method used to generate the random allocation sequence | 8 |
|  | 8b | Type of randomisation; details of any restriction (such as blocking and block size) | 8 |
| Allocation concealment mechanism | 9 | Mechanism used to implement the random allocation sequence (such as sequentially numbered containers), describing any steps taken to conceal the sequence until interventions were assigned | 8 |
| Implementation | 10 | Who generated the random allocation sequence, who enrolled participants, and who assigned participants to interventions | 7, 8 |
| Blinding | 11a | If done, who was blinded after assignment to interventions (for example, participants, care providers, those assessing outcomes) and how | Not applicable |
|  | 11b | If relevant, description of the similarity of interventions | Not applicable |
| Statistical methods | 12a | Statistical methods used to compare groups for primary and secondary outcomes | 8, 11-13 |
|  | 12b | Methods for additional analyses, such as subgroup analyses and adjusted analyses | Supplemental Material 2, 4-5, 13 |

| **Results** | | | |
| --- | --- | --- | --- |
| Participant flow (a diagram is strongly recommended) | 13a | For each group, the numbers of participants who were randomly assigned, received intended treatment, and were analysed for the primary outcome | 13-14, Supplemental material p. 18 |
|  | 13b | For each group, losses and exclusions after randomisation, together with reasons | 13-14, Supplemental material p. 18 |
| Recruitment | 14a | Dates defining the periods of recruitment and follow-up | 7 |
|  | 14b | Why the trial ended or was stopped | 7 |
| Baseline data | 15 | A table showing baseline demographic and clinical characteristics for each group | 14 |
| Numbers analysed | 16 | For each group, number of participants (denominator) included in each analysis and whether the analysis was by original assigned groups | 13-14 |
| Outcomes and estimation | 17a | For each primary and secondary outcome, results for each group, and the estimated effect size and its precision (such as 95% confidence interval) | 14-15, 17 |
|  | 17b | For binary outcomes, presentation of both absolute and relative effect sizes is recommended | Not applicable |
| Ancillary analyses | 18 | Results of any other analyses performed, including subgroup analyses and adjusted analyses, distinguishing pre-specified from exploratory | Supplemental Material 2-16 |
| Harms | 19 | All important harms or unintended effects in each group (for specific guidance see CONSORT for harms) | 13-14, Supplemental Material p.17 |

| **Discussion** | | | |
| --- | --- | --- | --- |
| Limitations | 20 | Trial limitations, addressing sources of potential bias, imprecision, and, if relevant, multiplicity of analyses | 22 |
| Generalisability | 21 | Generalisability (external validity, applicability) of the trial findings | 20-22 |
| Interpretation | 22 | Interpretation consistent with results, balancing benefits and harms, and considering other relevant evidence | 19-22 |
| **Other information** | | |  |
| Registration | 23 | Registration number and name of trial registry | Not applicable (non-CTIMP study) |
| Protocol | 24 | Where the full trial protocol can be accessed, if available | Not applicable |
| Funding | 25 | Sources of funding and other support (such as supply of drugs), role of funders | 24 |

Citation: Schulz KF, Altman DG, Moher D, for the CONSORT Group. CONSORT 2010 Statement: updated guidelines for reporting parallel group randomised trials. BMC Medicine. 2010;8:18.
© 2010 Schulz et al. This is an Open Access article distributed under the terms of the Creative Commons Attribution License (<http://creativecommons.org/licenses/by/2.0>), which permits unrestricted use, distribution, and reproduction in any medium, provided the original work is properly cited.

*We strongly recommend reading this statement in conjunction with the CONSORT 2010 Explanation and Elaboration for important clarifications on all the items. If relevant, we also recommend reading CONSORT extensions for cluster randomised trials, non-inferiority and equivalence trials, non-pharmacological treatments, herbal interventions, and pragmatic trials. Additional extensions are forthcoming: for those and for up-to-date references relevant to this checklist, see [www.consort-statement.org](http://www.consort-statement.org).

**Supplementary Table 2.** A CONSORT checklist for the current study. 'Not applicable' indicates categories that do now apply to the current study, as this was a non-Clinical Investigation of an Investigational Medicinal Product (non-CTIMP) study. The table is provided for the clarity of reporting.
